# Another challenge in malaria elimination efforts: the increase of malaria among adults after the implementation of long-lasting insecticide-treated nets (LLINs) in Dielmo, Senegal

**DOI:** 10.1186/s12936-018-2536-6

**Published:** 2018-10-25

**Authors:** Amélé N. Wotodjo, Souleymane Doucoure, Nafissatou Diagne, Fatoumata Diene Sarr, Philipe Parola, Jean Gaudart, Cheikh Sokhna

**Affiliations:** 1 UMR VITROME (Vecteurs-Infections Tropicales et Méditerranéennes) Campus International IRD-UCAD, Dakar, Senegal; 2 Aix Marseille Univ, IRD, AP-HM, SSA, VITROME, IHU-Méditerranée Infection, Marseille, France; 3 Unité d’Épidémiologie des maladies Infectieuses, Institut Pasteur de Dakar, Dakar, Senegal; 4 Aix Marseille Univ, IRD, INSERM, AP-HM, SESSTIM, BioSTIC, Marseille, France

**Keywords:** Malaria morbidity, Malaria resurgences, Adults, LLINs, Dielmo, Senegal

## Abstract

**Background:**

The widespread use of artemisinin-based combination therapy (ACT) and long-lasting insecticide-treated nets (LLINs) has led to an impressive decrease of malaria burden these recent years in Africa. However, some new challenges about the future of malaria control and elimination efforts have appeared. Among these challenges, the loss and—or—the only partial acquisition of anti-*Plasmodium* immunity among exposed populations lead to an increase of the age at risk of malaria. Indeed, older children and adults may become more vulnerable to malaria. Studies about malaria among adults seemed, therefore, important. This study investigated the evolution of malaria morbidity in adults of Dielmo (Senegal) before and after the implementation of LLINs.

**Methods:**

From August 2007 to July 2015, a longitudinal study involving adults above 15 years old was carried out in Dielmo, where ACT was introduced in June 2006 and LLINs in July 2008. In July 2011 and August 2014, all LLINs were renewed. The presence of each person in the village was monitored daily. Thick smears associated lately with rapid diagnosis test (RDT) and quantitative polymerase chain reaction methods were performed for all cases of fever. To assess malaria prevalence, thick smears and RDT were performed quarterly in all individuals. Malaria risks factors were assessed using negative binomial regression mixed-model based on person-trimester observations.

**Results:**

Malaria morbidity among adults has decreased significantly since the implementation of LLINs in Dielmo. However, malaria resurgences have occurred twice during the 7 years of LLINs use. During these malaria resurgences, the overall incidence of malaria among adults was similar to the incidence during the year before the implementation of LLINs (adjusted incidence rate ratio [95% CI] aIRR = 1.04 [0.66–1.64], p = 0.88 and aIRR = 1.16 [0.74–1.80], p = 0.52 during the first and the second malaria resurgence period, respectively). Younger adults were most vulnerable during these malaria upsurges as the incidence of malaria increased significantly among them (χ^2^ = 5.2; p = 0.02).

**Conclusion:**

Malaria among adults especially younger adults should deserve more attention in the areas where malaria was previously endemic as they became vulnerable probably because of the partial acquisition and—or—the loss of anti-*Plasmodium* relative immunity and the non regular use of LLINs.

## Background

Malaria burden decreased significantly in recent years in Africa through the widespread use of Artemisinin-based combination therapy (ACT) and long-lasting insecticide-treated nets (LLINs) associated with a combination of control interventions [1–3]. In the children < 5 years of age, and the pregnant women, that represent the population at risk, the prevalence and the incidence of the disease have collapsed dramatically compared to the period before the introduction of LLINs and ACT as first-line treatment of malaria. These considerable advances suggest the possibility of eliminating malaria in endemic countries [4]. However, this situation is changing the disease epidemiology, as suggested by an increase of the age at risk of malaria events [5]. Indeed, in former malaria endemic areas, older children and adults are becoming more vulnerable to malaria [2, 6, 7]. Several reasons may explain the increase of the age at risk. The compliance to LLINs use among older children and adults, and their behaviour during *Anopheles* biting time, have been incriminated [2, 7–9]. Further, the decrease of human exposure to malaria parasites due to the use of LLINs is reducing anti-*Plasmodium* immunity both in children and adults [5, 10–12]. The increase of the age at risk of malaria could maintain malaria residual transmission and generate serious concerns about the future of malaria elimination efforts [2, 7]. This is all the more important if consider that malaria controls and preventive measures have most often targeted children and pregnant women [13].

Because of these concerns, it seemed important to study malaria among adults in order to assess and adapt the current control tools. Studies of malaria in adults remain scarce, and new data on this topic are needed in the current context of the fall of malaria in some areas [1]. Therefore, an active monitoring of the population at risk is important in order to avoid malaria resurgences and to assess eventual new risk factors. The aim of this study was to investigate the evolution of malaria morbidity among adults of Dielmo (Senegal) between August 2007 and July 2015, after ACT was introduced in the village in June 2006 and LLINs were offered to all villagers in July 2008 and then renewed in July 2011 and August 2014. This study describes and analyses the change in malaria morbidity, prevalence and identifies the disease risks factors among adults aged at least 15 years old after the implementation of LLINs in the village of Dielmo.

## Methods

### Setting: Dielmo site

The Dielmo research site has been described in detail elsewhere [14]. The village is located in a Sudan-savannah region of central Senegal, 280 km south-east of Dakar on the marshy bank of the Nema, a small stream which allowed the persistence of anopheline breeding sites year-round. Since June 1990, a long-term malaria research project has been conducted among the population of Dielmo. Malaria transmission was continuous over the years from the beginning of the project until 2009, when transmission became seasonal. The epidemiology of malaria has changed significantly in this village, from holoendemic in 1990 to hypoendemic since 2010 [15]. In 2014, there were 45 concessions with approximately 450 inhabitants, including 245 adults aged at least 15 years.

### Participants and procedures

The inhabitants of Dielmo willing to participate at the project were involved in a longitudinal follow-up including: (i) monitoring of all episodes of fever, and, (ii) repeated quarterly cross-sectional surveys to document malaria prevalence and LLIN use. Written informed consent was obtained from all participants. The study was approved by the Ministry of Health of Senegal, the assembled village population and the National Ethics Committee of Senegal.

#### Medical surveillance of fever episodes

Body temperature was systematically recorded in adults in case of suspected fever or fever-related symptoms. In case of fever, patients were referred to the project health centre, which was open 24 h/day, 7 days/week. Thick smears stained with Giemsa were performed to determine the presence of the malaria parasite by using light microscopy. Episodes of fever were attributable to *Plasmodium falciparum* malaria clinical attacks, when parasite density was higher than an age-dependent threshold [16]. Malaria clinical attacks were treated with the combination artesunate plus amodiaquine since June 2006. From 2011 onwards, the diagnostic and treatment policy were modified to maximize efforts to limit malaria transmission. The RDT and PCR were then combined with the thick smear to improve disease diagnosis. Artesunate plus amodiaquine were systematically given to all patients with fever associated with malaria parasites, detected by at least one of these three diagnostic tools regardless of age and parasite density.

All participating households were visited daily. The presence or absence in the village of each enrolled household member was monitored and the location of the absent member was reported. Malaria morbidity was then assessed by the estimation of the incidence rate. For each period of the study, the malaria clinical attack incidence rate was calculated as the ratio of the number of clinical malaria attack recorded, divided by the number of person-days of follow-up during a given period.

#### Malaria prevalence cross-sectional surveys

To assess asymptomatic carriage and malaria prevalence each year, cross-sectional surveys were conducted quarterly, with two surveys during the dry season and two in the rainy season. Thick smears and RDT (since May 2011) were performed in all individuals enrolled in the Dielmo project who were present in the village during the survey.

#### Quarterly LLINs repeat cross-sectional surveys

LLINs (Permanet^®^ 2.0) were introduced for the first time in the village in July 2008 and have been offered to all villagers. In July 2011 and August 2014, all LLINs were renewed. In Dielmo, nets were distributed freely by sleeping place and the coverage is 100% with the slogan “one bed, one net”.

Simultaneously with the introduction of LLINs, repeat home-based surveys have been carried out to assess their use. Each participating household was visited quarterly, in the morning by two technicians, who verified ownership of nets and recorded whether the nets were hung above the bed the preceding night. They also administered a short questionnaire to household members about LLINs use. Individuals were asked if they had used nets the night preceding the visit, and whether they always or sometimes or never, used nets. Net ownership and it use were assessed per inhabitant of Dielmo present during the survey. All collected data were entered into the 4D software version 2004.5.

### Study population

This study focused on person-trimester observations covering the period before and after LLINs implementation, from August 2007 to July 2015. All adults of Dielmo who were enrolled in the project during this period and who had spent at least 30 days in the quarter in Dielmo were included in the analysis.

### Outcome and independent variables definition

The study was conducted in a period of 8 years from August 2007 to July 2015 and was divided in two periods: (i) a first period of 1 year (August 2007 to July 2008) corresponding to the time before LLINs implementation in Dielmo, which is used as a year of control; (ii) a second period of 7 years from August 2008 to July 2015 corresponding to the time of LLINs use in Dielmo.

Malaria clinical attacks were grouped into 32 quarters over 8 years (August–October, November–January, February–April and May–July of each year, previously defined). The analysis was thus based on person-trimester observations. The outcome variable was the number of malaria attacks per adult per quarter.

Rainfall was also measured each month of the study period. The following variables were analysed: (i) age group (15–19 years, 20–29 years, 30–44 years, 45–59 years and 60 years and older); (ii) rainfall; (iii) sex; (iv) pregnancy; (v) being quarterly resident (having spent at least 75% of the trimester’s days in Dielmo); (vi) being born in Dielmo; and (vii) the year of the use of LLINs.

Each variable was analysed separately using bivariate analysis to assess the association with malaria risk. Random-effect negative binomial regression models were used to analyse clinical malaria episodes and random-effect Poisson model was used to analyse the risk of malaria according to age group, taking into account the interdependence of successive observations in the same individuals. The days of monitoring for each person per quarter were controlled as the exposure variable. Variables that were p < 0.2 in bivariate analyses were integrated in multivariate analyses [17]. The significance level was fixed at p = 0.05 in the final model. The χ^2^ test for incidence rates was used to compare the incidence rate for each year. Analyses were performed using Stata Software, version 11.0 (College Station, Texas, USA).

## Results

### Description of participants

There were 187 and 243 adults enrolled in the project who had spent at least 1 month in the trimester in Dielmo during the first (August–October 2007) and the last quarter (May–July 2015) of the study respectively. A total of 6558 person-trimester observations (551,650 person-days) corresponding to 347 individuals aged from 15 to 103 years old with a mean of 38 years old and a proportion of 58% of women were analysed. Among these observations, 168 malaria clinical attacks were noted from whom 164 (2.5% of 6558 persons) were related to individuals who had at least one malaria attack per quarter during the study period and 6394 (97.5%) observations were related to adults who had no malaria attacks. The number of malaria clinical attacks varied from 1 to 3 attacks per adult per quarter.

### Incidence of malaria clinical attacks over the study period

**Fig. 1:**
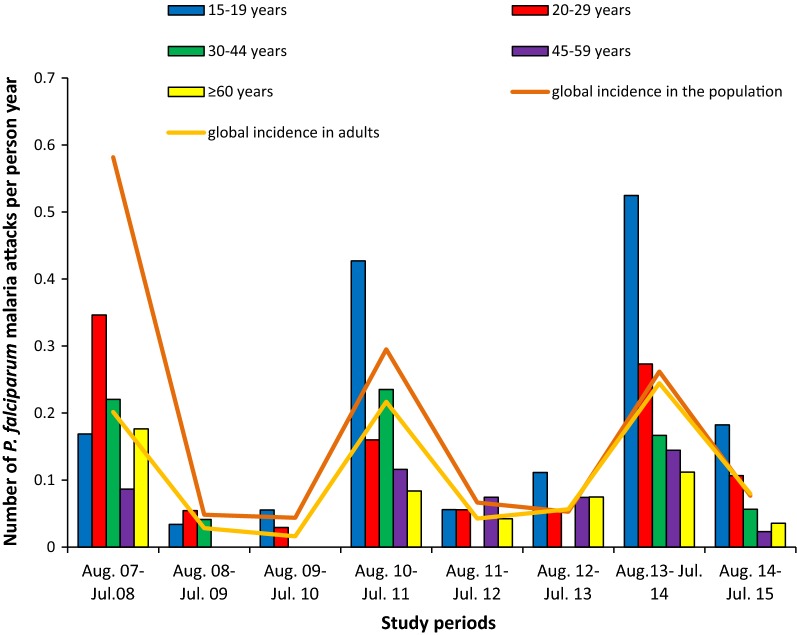
Incidence of malaria clinical attacks over the study period among adults in Dielmo

Malaria decreased consequently after the implementation of LLINs in Dielmo. However, adults became more vulnerable to malaria since the implementation of LLINs especially during malaria resurgence periods. Indeed, during the year before nets implementation, only 34% (0.20 attacks per person per year among adults compared with 0.58 in the Dielmo population, adults representing approximately 54% of the study population) of malaria incidence rate and only 18% (34/189) of malaria cases occurred among adults, whereas since the implementation of nets, approximately the same malaria incidence was observed among adults and children (0.06 attacks per person per year among adults and children during the fourth and fifth years of LLINs implementation) (Fig. 1).

A rise of malaria has occurred at the third year of nets use, and the malaria incidence increased from 0.02 attacks per adult per year during the second years of nets implementation to 0.22 attacks per adult per year. In response to the increase in malaria, all the LLINs were renewed in July 2011. Following that, malaria consequently decreased from 0.22 attacks per adult per year recorded during the upsurge to 0.04 and 0.06 attacks per adult per year during the fourth and the fifth year of nets implementation respectively (p < 0.001).

A second upsurge occurred during the sixth year of the nets implementation corresponding to the third year of the renewal of LLINs, and the malaria incidence shifted to 0.24 attacks per adult per year (p < 0.001). This led to a second renewal of all LLINs in Dielmo in August 2014 and the malaria attack decreased again to 0.08 attacks per adult per year in the following year corresponding to the 8 year of the nets implementation (p < 0.001) (Fig. 1). Overall, the mean incidence of malaria was 0.08 attacks per adult per year during the malaria decreased periods when nets were implemented, and was 0.24 during malaria upsurge periods and was 0.20 during the year before nets implementation.

### Malaria incidence according to age groups of adults

Comparing with the year before nets implementation, malaria incidence has increased significantly among age group 15–19 years during malaria upsurge periods (χ^2^ = 5.2; p = 0.02) (Fig. 1). Among the individuals aged 15–19 years, the incidence of malaria was 0.17 attacks per person per year during the year before nets implementation, whereas during malaria upsurges period, malaria incidence increased to 0.43 and 0.52 attacks per person per year the first and the second malaria upsurge periods respectively. Among older adults, no significant difference was observed during malaria upsurge periods comparing with the year before nets implementation (Fig. 1).

During the year before net implementation, no difference of malaria incidence was observed according to age groups (compared to adults aged 15–19 years, incidence rate ratio [95% CI] IRR = 1.85 [0.63; 5.44] p = 0.27; IRR = 1.31 [0.45; 3.86] p = 0.62; IRR = 0.53 [0.12; 2.27] p = 0.39, IRR = 1.11 [0.29; 4.27] p = 0.88 respectively for adults aged 20–29 years 30–44 years, 45–59 years and 60 years and older) whereas during malaria upsurge periods, younger adults were more vulnerable to malaria than the oldest (compared to younger adults aged 15–19 years, IRR = 0.44 [0.25; 0.78] p = 0.005; IRR = 0.43 [0.25; 0.73] p = 0.002; IRR = 0.28 [0.14; 0.56] p < 0.001, IRR = 0.22 [0.08; 0.55] p = 0.001 respectively for adults aged 20–29 years 30–44 years, 45–59 years and 60 years and older).

### Factors associated with the risk of malaria clinical attacks

Table 1 describes both characteristics according to malaria attacks and the results of the bivariate and multivariate analyses. In bivariate analysis, compared with the year before nets implementation, the risk of malaria attacks among adults was the same during the third (the first malaria upsurge period) and sixth year (the second malaria upsurge period) after the nets implementation (IRR = 1.09 [0.69; 1.73] p = 0.71; IRR = 1.25 [0.81; 1.95] p = 0.32 respectively). Older adults had fewer clinical malaria attacks compared with younger adults aged 15–19 years (IRR = 0.59 [0.39; 0.89] p = 0.013; IRR = 0.44 [0.29; 0.66] p < 0.001; IRR = 0.32 [0.20; 0.53] p < 0.001, IRR = 0.34 [0.18; 0.62] p < 0.001, respectively for adults aged 20–29 years 30–44 years, 45–59 years and 60 years and older). Being born at Dielmo, being quarterly resident of Dielmo, rainfall and the sex were not significantly associated with malaria risk. Pregnancy was associated with malaria risk [(IRR = 2.70 [1.43; 5.12] p = 0.02].

**Table 1:** Socio-demographic and other characteristics according to malaria attacks and results of random-effect negative binomial regression models exploring factors associated with malaria clinical cases (n = 6558)

Characteristics	Subcategory	Number of observations n = 6558 n (%)	Malaria cases		Bivariate analysis		Multivariate analysis	
			No, n = 6394 n (%)	Yes, n = 164 n (%)	IRR (95% CI)	*P* value	aIRR (95% CI)	*P*-value
*Socio-demographic characteristics*								
Age group								
	15–19 years (ref)	1227 (18.71)	1170 (18.30)	57 (34.76)	1		1	
	20–29 years	1363 (20.78)	1326 (20.74)	37 (22.56)	0.59 (0.39–0.89)	0.013	0.68 (0.45–1.03)	0.07
	30–44 years	1771 (27.01)	1735 (27.13)	36 (21.95)	0.44 (0.29–0.66)	< 0.001	0.45 (0.30–0.69)	< 0.001
	45–59 years	1378 (21.01)	1357 (21.22)	21 (12.80)	0.32 (0.20–0.53)	< 0.001	0.33 (0.20–0.54)	< 0.001
	60 years and over	819 (12.49)	806 (12.61)	13 (7.93)	0.34 (0.18–0.62)	< 0.001	0.33 (0.18–0.22)	< 0.001
Sex								
	Male (ref)	2780 (42.39)	2699 (42.21)	81 (49.39)	1		1	
	Female	3778 (57.61)	3695 (57.79)	83 (50.61)	0.76 (0.55–1.05)	0.09	0.89 (0.65–1.22)	0.47
Resident/quarter								
	No (ref)	770 (11.74)	753 (11.78)	17 (10.37)	1			
	Yes	5788 (88.26)	5641 (88.22)	147 (89.63)	1.17 (0.71–1.95)	0.53		
Born at Dielmo								
	No (ref)	1969 (30.02)	1923 (30.08)	46 (28.05)	1			
	Yes	6558 (69.98)	4471 (69.92)	118 (71.95)	1.11 (0.78–1.58)	0.56		
Pregnancy								
	No (ref)	3567 (94.42)	3495 (94.59)	72 (86.75)	1			
	Yes	211 (5.58)	200 (5.41)	11 (13.25)	2.70 (1.43–5.12)	0.002		
Year of the use of LLINs (1)								
	Year before nets implementation (ref)	756 (11.53)	724 (11.32)	32 (19.51)	1		1	
	First year of the use of LLINs	771 (11.76)	766 (11.98)	5 (3.05)	0.14 (0.06–0.37)	< 0.001	0.14 (0.05–0.35)	< 0.001
	Second year of the use of LLINs	818 (12.47)	815 (12.75)	3 (1.83)	0.08 (0.02–0.27)	< 0.001	0.08 (0.02–0.25)	< 0.001
	Third year of the use of LLINs	829 (12.64)	788 (12.32)	41 (25)	1.09 (0.69–1.73)	0.71	1.04 (0.66–1.64)	0.88
	Fourth year of the use of LLINs	808 (12.32)	800 (12.51)	8 (4.88)	0.22 (0.10–0.47)	< 0.001	0.21 (0.09–0.44)	< 0.001
	Fifth year of the use of LLINs	839 (12.79)	828 (12.95)	11 (6.71)	0.29 (0.14–0.57)	< 0.001	0.27 (0.14–0.56)	< 0.001
	Sixth year of the use of LLINs	873 (13.13)	828 (12.90)	48 (29.27)	1.25 (0.81–1.95)	0.32	1.16 (0.74–1.80)	0.52
	Seventh year of the use of LLINs	864 (13.17)	848 (13.26)	16 (9.76)	0.41 (0.22–0.74)	0.003	0.39 (0.21–0.70)	0.002
Rainfall								
					1.0001 (0.9996–1.0007)	0.60	1.0002 (0.9997–1.0007)	0.57

After adjusting for potential covariates such as age, sex, rainfall and days of monitoring of each person per quarter, no difference in malaria risk was found during the third year (the first malaria upsurge period) and sixth year (the second malaria upsurge period) after nets implementation compared with the year before nets implementation indicating that the risk of malaria during these years (third and sixth year) of nets implementation equal the risk of malaria before nets implementation in Dielmo (aIRR = 1.04 [0.66; 1.64], p = 0.88 and aIRR = 1.16 [0.74; 1.80], p = 0.52, respectively for the third and sixth year after nets implementation). The malaria risk remained lower among older adults compared to younger adults aged 15–19 old except for age group 20–29 years for whom no significant difference of malaria risk was observed (aIRR = 0.68 [0.45; 1.03] p = 0.07; aIRR = 0.45 [0.30; 0.69] p < 0.001; aIRR = 0.33 [0.20; 0.54] p < 0.001; aIRR = 0.33 [0.18; 0.62] p < 0.001, respectively for adults aged 20–29 years, 30–44 years, 45–59 years and 60 years and older). Rainfall remained not significantly associated with a risk of having malaria (aIRR = 1.0002 [0.9997; 1.0007], p = 0.45).

### Malaria prevalence

**Fig. 2:**
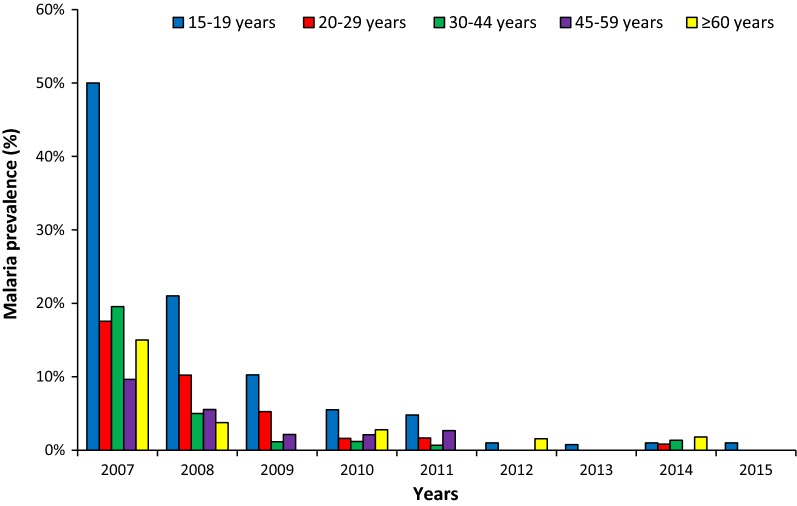
Malaria prevalence among adults in Dielmo from 2007 to 2015

Since the implementation of the nets, the prevalence of malaria among adults has decreased significantly from 23% in 2007 (before LLIN) to 0.9% in 2014. During the upsurge periods, the prevalence did not increase and was 2.5% and 0.2% in 2010 and 2013, respectively. According to the age group, the prevalence was however slightly higher in youngest adults than oldest over the study period (Fig. 2). Indeed, during 2008 and 2009, the mean of malaria prevalence was 11.7% among individuals aged 15–29 years and 2.9% among those aged 30 years and over. During the upsurge period in 2010, the mean prevalence was 3.6% among individuals aged 15–29 years and 2.0% among those aged 30 years and over.

### Entomological inoculation rate (EIR)

The EIR decreased from 96.1 infective bites per person per year in 2008 to only 45.6 infective bites per person per year in 2009. However, it increased in 2010 during the first upsurge period to 88.7 infective bites per person per year. In 2013, during the second malaria upsurge, the EIR increased from 7.6 infective bites per person per year in 2012 to 43.1 infective bites per person per year. In 2014 and 2015, the EIR remained low and was 18.2 and 2.8 infective bites per person per year, respectively.

### Net use

**Fig. 3:**
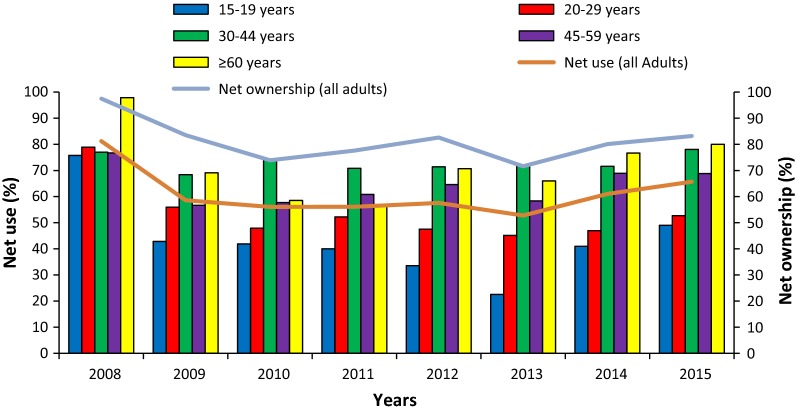
LLINs ownership and its use according to the year among adults by age groups

The overall use of nets was slightly higher among older adults than young adults aged 15–29 years except 2008 (Fig. 3). During the first malaria upsurge period in 2010, the use of nets was significantly low among young adults aged 15–29 years and adults aged 45–59 years compared with adults aged 30–44 years old (OR = 0.07[0.02; 0.25] p < 0.001; OR = 0.06[0.02; 0.24] p < 0.001; OR = 0.23[0.06; 0.85] p = 0.027 respectively for adults aged 15–19 years; 20–29 years and 45–59 years). During the second malaria resurgence period in 2013, older adults aged 30–44 years used frequently their nets than young adults aged 15–29 years (OR = 0.15[0.004; 0.61] p < 0.001 and OR = 0.09[0.02; 0.31] p < 0.001, respectively for adults aged 15–19 years and 20–29 years compared with adults aged 30–44 years).

Except 2008, the first year of the implementation of nets, where the use of nets was the highest (81%), no significant difference was noted for the use of nets according to the year of nets use. The use of nets has then decreased from 81% in 2008 to its lowest in 2013 with only 53% of nets use (Fig. 3). During the periods of malaria upsurge, the use of nets was 57% and 53% in 2010 and 2013, respectively. However, the use of nets during these upsurge periods was not significantly different from other years of nets use except in 2008.

The ownership of net was also the highest in 2008 with 97.5%; it decreased slightly to 83.5% the second year of net implementation. The ownership of net reached its lowest level in 2013 with 71.6% of net ownership. Over the study period, no important difference in net ownership was observed except in 2008 (Fig. 3).

## Discussion

This study has shown the evolution of clinical malaria among adults before and after the introduction of LLINs in the village of Dielmo. The efficacy of ACT together with LLINs to reduce malaria incidence was observed in this study after nets implementation in Dielmo in July 2008. This result is in line with the trend observed in many endemic areas [18, 19].

Unfortunately, two malaria resurgences occurred during 7 years of nets universal coverage in Dielmo and the disease incidence has increased significantly among younger adults when comparing with the period before the implementation of LLINs. This observation underlines the fragility of current malaria control strategies based mainly on LLINs use. The relaxation of the population to the regular use of LLINs constitutes one of the barriers to the effectiveness of this tool [19, 20]. Indeed, despite LLIN implementation at the Dielmo village level, malaria resurgences were observed in young adults who represent the population at risk during these upsurges. This situation was probably due to the low level of LLINs use in younger adults (15–19 years) compared to the rest of the population.

The non-regular use of nets among younger adults could be due to their behaviour as they tended to stay much longer outside at night. This outdoor behaviour has been shown previously in some studies as malaria risk factor [2, 8]. In Dielmo after the implementation of LLINs, the residual vector populations of *Anopheles gambiae* sensu lato and *Anopheles funestus* had an increased preference to bite outdoors [21]; thus, spending time out lately at night remained malaria risk factor in this village [9]. Another explanation of the shift of malaria risk age was that children often slept under LLINs with their mother, while adult men specially slept later and not often used their nets [22]. Some studies in Asia explained the increase of malaria attacks in adult men by forestry and farmings activities and outdoor sleeping behaviour for crops protection; thus raising their level of exposure to vector bites, compared to others groups of the population [2, 7]. In South Africa and Swaziland, where malaria has decreased drastically, the incidence of malaria was highest among men and this was associated with their outdoor activities [8, 23]. According to gender, no difference in malaria risk was noted in this study.

The vulnerability of adults especially the youngest could also be explained by a poor acquired immunity against malaria. One year before the implementation of nets in Dielmo, all adults were almost at same risk of malaria, whereas after the implementation of nets especially during malaria upsurge periods, younger adults became more vulnerable to malaria. It has to be noted that these younger adults were almost children when nets were implemented in the village. They were conceivably less susceptible to develop an immunity against malaria during their adulthood, since LLINs act by reducing human-vector contact, and thereby reducing their exposure to *Plasmodium* parasites. Nets use could decrease immunity against malaria among population who have often used LLINs [24, 25]. It was demonstrated by previous studies that the decrease of malaria transmission could shifted the age risk of malaria and thus increase the age of immunity against malaria [5, 10]. During the study period, malaria prevalence decreased greatly, showing the scarcity of asymptomatic cases because of the probable non-acquired and or loss of immunity [10–12] as almost all new infections became symptomatic. Adults of Dielmo were highly protected in the past against malaria because of their acquired immunity; this immunity has decreased recently with the introduction of nets [10, 12], especially in younger adults. This situation was observed in other areas [5, 6, 24]. Studies in Kenya showed that the mean age of people with clinical attacks increased steadily as exposure to mosquito declined [5], indicating a probable loss of immunity against malaria and the shift in the age of malaria risk. Despite the probable loss or late acquisition of anti-*Plasmodium* immunity, no severe malaria was observed during the study period, perhaps because of the close monitoring of the population.

The important reduction of malaria in Dielmo after the implementation of LLINs was spoiled by the occurrence of two malaria resurgences. During these malaria resurgences, the overall incidence of malaria equals the incidence of malaria before the implementation of nets among adults while malaria increased significantly among younger adults. Before the implementation of nets, the portion of malaria attacks among adults was only 18%, and since 2010, this portion has been approximately 50%. This observation underlines the fragility of malaria elimination efforts among adults and then their vulnerability. The current prevention methods target mostly the children under five and pregnant women and tend to reduce the incidence of malaria in these risk groups and to shift the burden of the disease to older children and adults [2]. During the time of net use, except for malaria resurgence periods in adults, the overall malaria incidence remained significantly low in Dielmo [26].

## Conclusions

These results highlight the need to take into account adults in the fight against malaria, and possible elimination. Malaria in adults, especially young adults, should deserve more attention in the areas where malaria was previously endemic, as adults become vulnerable probably because of the reduced acquisition and/or the loss of anti-*Plasmodium* immunity and the non-regular use of their nets. Awareness campaign and monitoring of the use of nets remained crucial to avoid malaria resurgences.
